# Abscisic Acid Regulates the Occurrence and Recovery of the Striped Leaf Phenotype in Response to Lacking Light at the Base of Sheath in Rice by Modulating Carbohydrate Metabolism

**DOI:** 10.3390/plants13152090

**Published:** 2024-07-28

**Authors:** Jiasheng Chen, Shaowen Yang, Ming Fu, Ying He, Hanlai Zeng

**Affiliations:** MOA Key Laboratory of Crop Ecophysiology and Farming System in the Middle Reaches of the Yangtze River, College of Plant Science and Technology, Huazhong Agricultural University, Wuhan 430070, China; jiashengchen@webmail.hzau.edu.cn (J.C.); yangsw@webmail.hzau.edu.cn (S.Y.); fuming2016@webmail.hzau.edu.cn (M.F.)

**Keywords:** rice, shading of the leaf sheath, intermittent leaf discoloration, glycometabolism, abscisic acid biosynthesis, sucrose

## Abstract

Rice B03S mutants with intermittent leaf discoloration were developed from the photoperiod- and thermosensitive genic male sterile (PTGMS) rice line Efeng 1S. After these plants were deeply transplanted, the new leaves manifested typical stripe patterns. In this study, deep and shallow transplantation of B03S was carried out, and aluminum shading was performed directly on the leaf sheath. It was determined that the reason for the appearance of the striped leaf trait was that the base of leaf sheath lacked light, at which time the sheath transformed from the source organ to the sink organ in rice. To elucidate the related metabolic changes in glycometabolism and abscisic acid (ABA) biosynthesis and transcriptional regulation in the leaf sheath, ultra-performance liquid chromatography/tandem mass spectrometry (UPLC-MS/MS) combined with transcriptome and real-time quantitative PCR (qPCR) validation were used for analysis after deep and shallow transplantation. The result indicates that the leaf sheath may need to compete with the new leaves for sucrose produced by the photosynthesis of old leaves in response to lacking light at the base of sheath. Moreover, the ABA content increases in the leaf sheath when the gene expression of *ABA2* and *AAO1* is upregulated at the same time, enhancing the plant’s resistance to the adverse condition of shading at the leaf sheath. Furthermore, exogenous spraying of B03S with ABA solution was carried out to help recovery under shading stress. The result indicates that the synthesis of endogenous ABA in the leaf sheath is reduced by spraying ABA. At the same time, ABA regulates sucrose metabolism by inhibiting the expression of the *SUS* gene. This allows for more sucrose synthesized by the old leaves to be transported to the new leaves, resulting an obvious recovery effect of the strip leaf character due to the re-balance of sugar supply and demand in B03S. These findings improve the understanding of the physiological function and metabolic mechanism of the rice leaf sheath, provide a theoretical basis for uneven leaf coloration in nature, and provide theoretical guidance for rice production via seedling transplantation or direct seeding.

## 1. Introduction

Rice (*Oryza sativa* L.) is one of the most important food crops in China and worldwide, contributing greatly to food security. More than 90% of the rice yield depends on leaf photosynthesis; thus, improving photosynthetic efficiency during grain production is an effective measure for increasing rice yield [1]. Photosynthesis is the key process of energy conversion and carbon assimilation from chloroplasts for plant growth and development. In plants, the developmental state of chloroplasts and the synthesis and degradation of chlorophyll are closely related to photosynthetic capacity [2]. Therefore, improving the understanding of chloroplast development and chlorophyll metabolism in food crops should contribute to improving yield.

The photosynthetic pigments of plants play a role in absorbing, transferring, and transforming light energy. Generally, the degree of greenness of leaves reflects their chlorophyll content, which is an important phenotypic index in rice cultivation and management. Leaf color is determined by the contents and proportions of various other photosynthetic or accessory pigments in addition to chlorophyll. Mutations that cause leaf color variation are common in plants such as Arabidopsis, wheat, rice, and maize [3,4,5,6]. In our previous research, a natural mutant of B03S with zebra leaf traits was obtained from the offspring of the photoperiod- and thermosensitive genic male sterile (PTGMS) rice line Efeng 1S. These leaf color mutants should help to facilitate the study of chloroplast structure and chlorophyll anabolic mechanisms and to enhance the understanding of photosynthesis mechanisms and photomorphogenesis [7].

In general, the mechanism of leaf color mutation can be divided into the following three types. First, mutations of related genes in the pathways of chlorophyll biosynthesis and degradation affect the normal metabolism of chlorophyll, thereby causing changes in the proportion of chloroplast pigments and in leaf color [8]. In the Arabidopsis *NOL/NYC1* double mutant with a mutation in the gene encoding chlorophyll b reductase, chlorophyll b cannot be degraded to chlorophyll a, resulting in an evergreen phenotype [9]. Second, mutations in genes related to chloroplast development, such as the *CSP41b* gene in rice, can lead to impaired chloroplast development and make leaves appear light green [10]. Third, mutations in genes related to heme and carotenoid biosynthesis often manifest as yellowing or light-colored leaves. In the rice mutant *ygl2*, the expression of the oxygenase gene *YGL2* significantly decreased, resulting in a yellow–green leaf phenotype [11]. In transgenic tobacco overexpressing two *PSY* genes encoding phytoene synthase, a key enzyme involved in carotenoid synthesis, changes in leaf morphology and pigmentation were detected [12,13]. Fourth, the differential expression of abscisic acid (ABA) synthesis genes, such as the 9-cis-epoxycarotenoid dioxygenase (NCED) and carotenoid cleavage dioxygenase (CCD) genes downstream of the carotenoid metabolic pathway, is generally induced by environmental factors to regulate physiological processes, leading to leaf albinism [14,15].

B03S is characterized by the loss of greenness in the leaf sheath and leaves, resulting in yellow–green intermittent stripes similar to zebra stripes. This mutant character is controlled by a pair of recessive genes and can be stably inherited. It has been proven that a direct cause of the occurrence of zebra leaf traits is the leaf sheath being hidden in the dark, which is usually caused by deep transplantation [16]. Furthermore, we revealed the physiological and metabolic mechanism wherein carotenoids flow to the lutein cycle under photooxidative stress, and the differentially abundant metabolites of zeaxanthin resulting from *OsZEP* expression are upregulated in the zebra striped leaves. In-depth study of the material can provide theoretical guidance for optimizing the technical parameters of rice production, such as determining the appropriate depth for the growth and development of rice in practical agricultural operations such as mechanical transplantation and determining the depth for direct seeding.

The rice leaf sheath is located between the stem and the leaf, but throughout the vegetative growth period, there is no true stem or culm development. The stem of rice is called the culm. The aboveground section of rice seedlings includes leaf blades, ligule/anricles, and sheaths, with leaf blades held up by tightly wrapped leaf sheaths [17]. Its main physiological functions include supporting and connecting leaves and stems subsequently. Besides its function in physical support, it accumulates carbohydrates before heading, and carbohydrate contents vary with position in a leaf sheath. Tsuno found that the upper part of the leaf sheath had high photosynthetic capacity, but the lower part had almost no photosynthetic capacity [18,19]. Thus, the leaf sheath of rice has a strong photosynthetic function and potentially contributes to yield. Although the genetics and physiology of B03S have been studied previously, the specific regulatory mechanism involved in the loss or recovery of greenness in leaves is still unclear. There was clear sugar deficiency in the zebra striped seedlings, and the expression of sugar transport-related genes decreased significantly in the leaves that lost greenness or exhibited yellowing. In addition, the characteristic pigment metabolic pathway associated with the zebra stripe trait is the carotenoid pathway, metabolites from which also serve as precursors for ABA synthesis [16]. Therefore, we hypothesized that ABA metabolic pathways and sucrose metabolic pathways play important roles in the formation and recovery of the loss-of-greenness trait in B03S. To test this hypothesis, metabolomic analysis targeting glucose and ABA metabolism was carried out for B03S sheaths using liquid chromatography–mass spectrometry (LC–MS/MS), and potential target genes were selected and validated at the gene expression level based on transcriptome analysis and real-time quantitative PCR (qPCR) in this study. These results combined with the results of the exogenous spray test showed that the ABA and sucrose metabolism pathways played a significant role in the formation and recovery of the zebra stripe phenotype in B03S leaves. Taken together, these findings provide new insights into the metabolic mechanisms underlying uneven leaf color distribution in plants.

## 2. Results

### 2.1. Dark Shading of the Leaf Sheath Induces the Loss of Greenness in the Leaves of B03S

**Figure 1:**
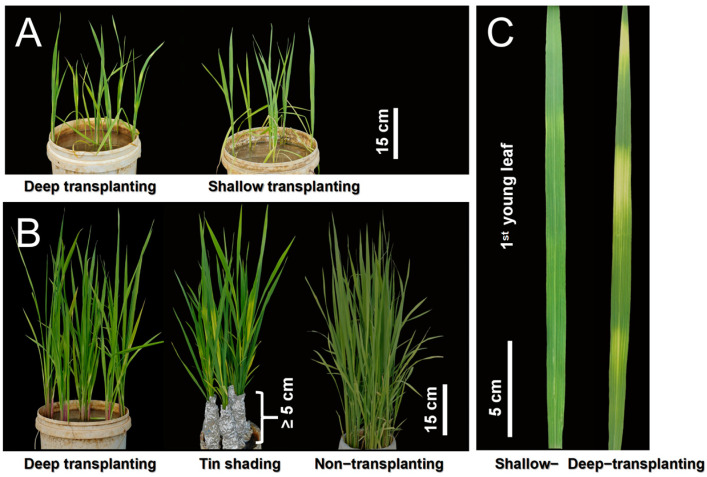
An obvious striped leaf phenotype occurred in B03S under the dark shading treatment of the leaf sheath. (**A**) Leaf phenotype of plants subjected to deep and shallow transplantation. (**B**) Leaf phenotypes of plants after covering with aluminum foil, non-transplantation, and deep transplantation. (**C**) Zebra trait phenotype in B03S leaves.

To obtain plants with striped leaves, B03S seedlings in the third-leaf and fifth-leaf stages were subjected to three different leaf sheath shading treatments, namely, shallow transplantation (ST), deep transplantation (DT), and aluminum foil covering. After five days of treatment, zebra stripes appeared on the new leaves of the plants in two groups including the DT group and the aluminum covering group (Figure 1A,B). The zebra stripe trait of B03S manifested as new leaf sheath bleaching and transverse and intermittent yellow–green stripe formation on the younger leaves, which continued for 35 days (Figure 1C). In the ST and non-transplanted groups, there were no loss-of-greenness-related stripes on the leaf sheaths or leaves of B03S (Figure 1A,B). Here, the zebra-stripe leaf pattern that arose during rice seedling transplantation was due to insufficient light for the leaf sheath in the mutant material B03S.

### 2.2. Metabolomic Analysis of the Leaf Sheath of B03S

According to the above findings, the zebra stripe leaf pattern appeared at 5 DAT, which occurred because of insufficient light for the leaf sheath. To examine the carbohydrate metabolism within the leaf sheath during this period, 4 cm leaf sheaths of B03S were subjected at 4 DAT (before the appearance of the zebra leaves) and 5 DAT (at the time of the new zebra leaf unfolding) in the deep transplantation and shallow transplantation groups to targeted metabolomic analysis of glycometabolism through UPLC–MS/MS.

**Figure 2:**
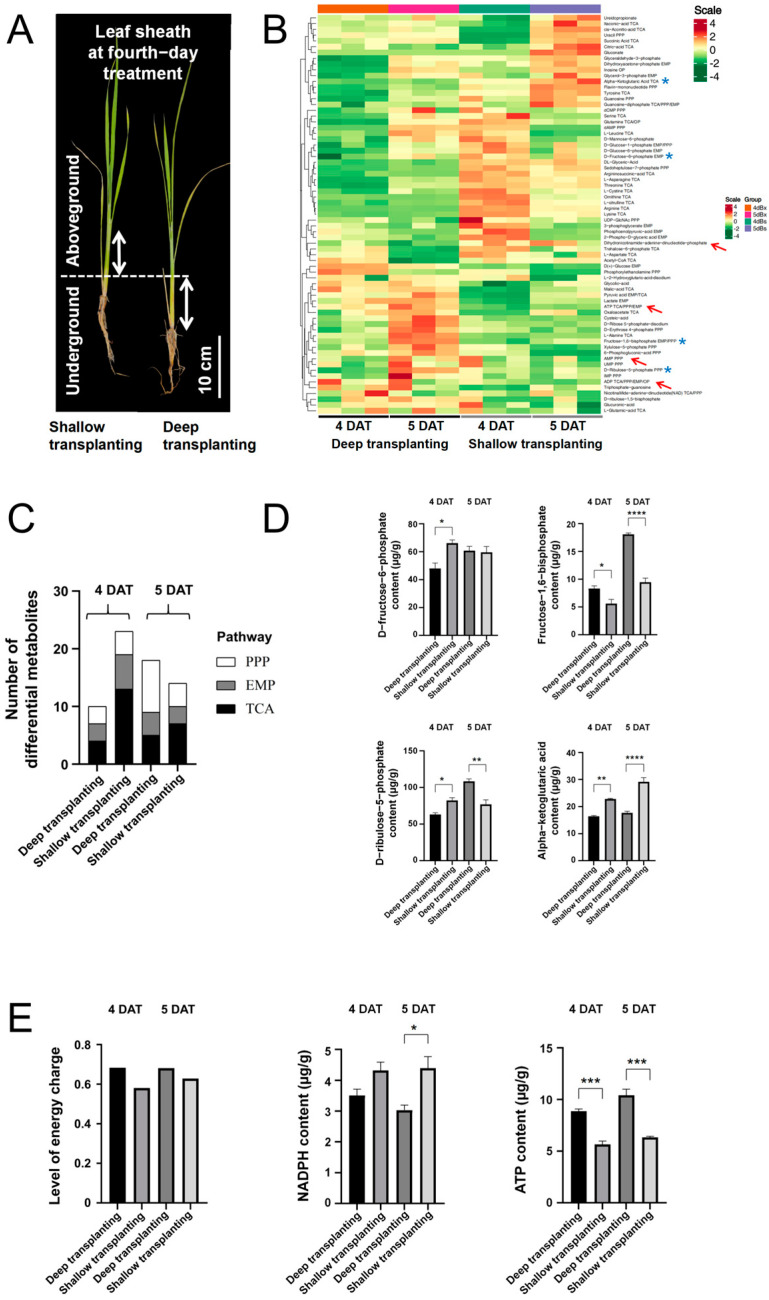
Differential analysis of glycometabolism in the leaf sheaths of B03S plants subjected to the dark shading treatment. (**A**) Phenotype and leaf sheath of plants subjected to deep and shallow transplantation at 4 DAT. (**B**) Heatmap analysis of the main differentially accumulated metabolites of the glycometabolism and energy metabolism pathways. The blue asterisk indicates representative metabolites from three different branching pathways. The arrow indicates metabolites related to NADPH and ATP. (**C**) The amounts of the main differentially accumulated metabolites in three different pathways. (**D**) The levels of four representative types of metabolites. (**E**) Metabolite content of NADPH and ATP. The energy charge was calculated as (ATP + 0.5 × ADP)/(ATP + ADP + AMP). The data are shown as the mean ± standard error (*n* = 3). The black asterisks indicate significant differences according to Student’s *t* test (*, *p* < 0.05; **, *p* < 0.01; ***, *p* < 0.001; ****, *p* < 0.0001).

The deep transplantation (DT) and shallow transplantation (ST) samples contained 4 cm sheaths of B03S from underground and aboveground parts, respectively. A total of 69 differentially accumulated metabolites related to glycometabolism were detected (Figure 2A,B), which included metabolites from the Embden–Meyerhof-Parnas pathway (EMP), tricarboxylic acid cycle pathway (TCA), and pentose phosphate pathway (PPP). Through cluster analysis of these different metabolites, a total of 37 energy metabolism intermediates with significantly increased contents were screened from the leaf sheaths subjected to ST, most of which were involved in normal growth, among which 9 metabolites were metabolites of the EMP pathway, 20 metabolites were of the TCA pathway, and 8 metabolites were of the PPP. A total of 28 energy metabolites whose content increased significantly were screened from leaf sheaths subjected to DT. Most of the metabolites in the leaf sheaths were involved in stress resistance, except for those involved in energy for growth maintenance. Among these metabolites, 7 were metabolites of the EMP, 9 were metabolites of the TCA pathway, and 12 were metabolites of the PPP (Figure 2C).

In the EMP, fructose-6-phosphate (F-6-P) and fructose-1,6-diphosphate (F-1,6-P) are key intermediate metabolites. At 4 DAT, the F-6-P content of the DT sheaths decreased by 28%, while the F-1,6-P content increased by 1.48-fold, compared with that of the ST sheaths during the corresponding period. Subsequently, the F-1,6-P content further increased by 1.91-fold at 5 DAT in the DT sheaths (Figure 2D). Ribulose-5-phosphate (R-5-P) is a key metabolite of the PPP. At 4 DAT, the R-5-P content in the DT sheaths decreased by 23% compared with that in the ST sheaths. In contrast, it increased 1.41-fold at 5 DAT in the DT sheaths (Figure 2D). The content of alpha-ketoglutaric acid, a key metabolite of the TCA pathway, in the DT sheaths was clearly lower than that in the ST sheaths, with decreases of 28% and 39% at 4 DAT and 5 DAT, respectively (Figure 2D).

The analysis of energy metabolites confirmed the above results. Adenosine triphosphate (ATP) is one of the most important end products of the EMP and TCA pathway. The ATP content in the DT leaf sheaths increased 1.57- to 1.64-fold compared with that in the ST sheaths (Figure 2E). The energy charge level of the leaf sheath significantly increased under DT (Figure 2E).

The reduced form of nicotinamide adenine dinucleotide phosphate (NADPH) is an important product of the PPP. The NADPH content in the DT leaf sheaths of DT was 18.76% to 30.98% lower than that in the ST leaf sheaths (Figure 2E). Therefore, we believe that in ST leaf sheaths, the main glycometabolism pathway for ATP production is the TCA pathway. However, in the DT leaf sheaths, the main glycometabolism pathways were the PPP for reducing NADPH and the EMP for faster and greater ATP generation.

### 2.3. Analysis of the Abscisic Acid Content in the Leaf Sheath of B03S

Previous research has shown that the carotenoid pathway is involved in the formation of zebra leaves [16]. Zeaxanthin is a metabolite that accumulates differentially in zebra striped leaves and is also a precursor for ABA synthesis.

**Figure 3:**
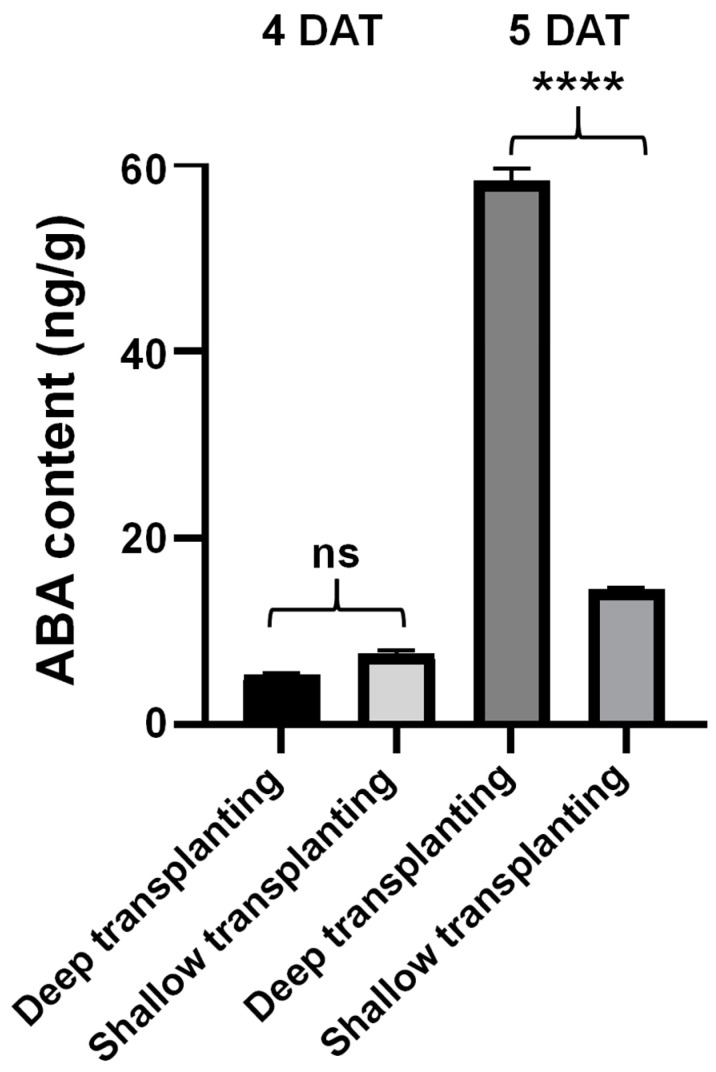
Differential analysis of ABA content in the leaf sheath of B03S. Black asterisks indicate significant differences according to Student’s *t* test (****, *p* < 0.0001); ns indicates no significant difference (*p* > 0.05).

Here, we focused on the accumulation of ABA in leaf sheaths after DT. The ABA contents were 5.36 ng/g and 58.21 ng/g at 4 DAT and 5 DAT, respectively, in the DT sheath. Notably, the ABA content at 5 DAT in the DT sheath was 3.99-fold greater than that at 5 DAT in the ST sheath (Figure 3). Therefore, the ABA content in the leaf sheaths significantly increased in B03S in response to DT stress.

### 2.4. Transcriptome Sequencing Analysis of the Leaf Sheath of B03S

**Figure 4:**
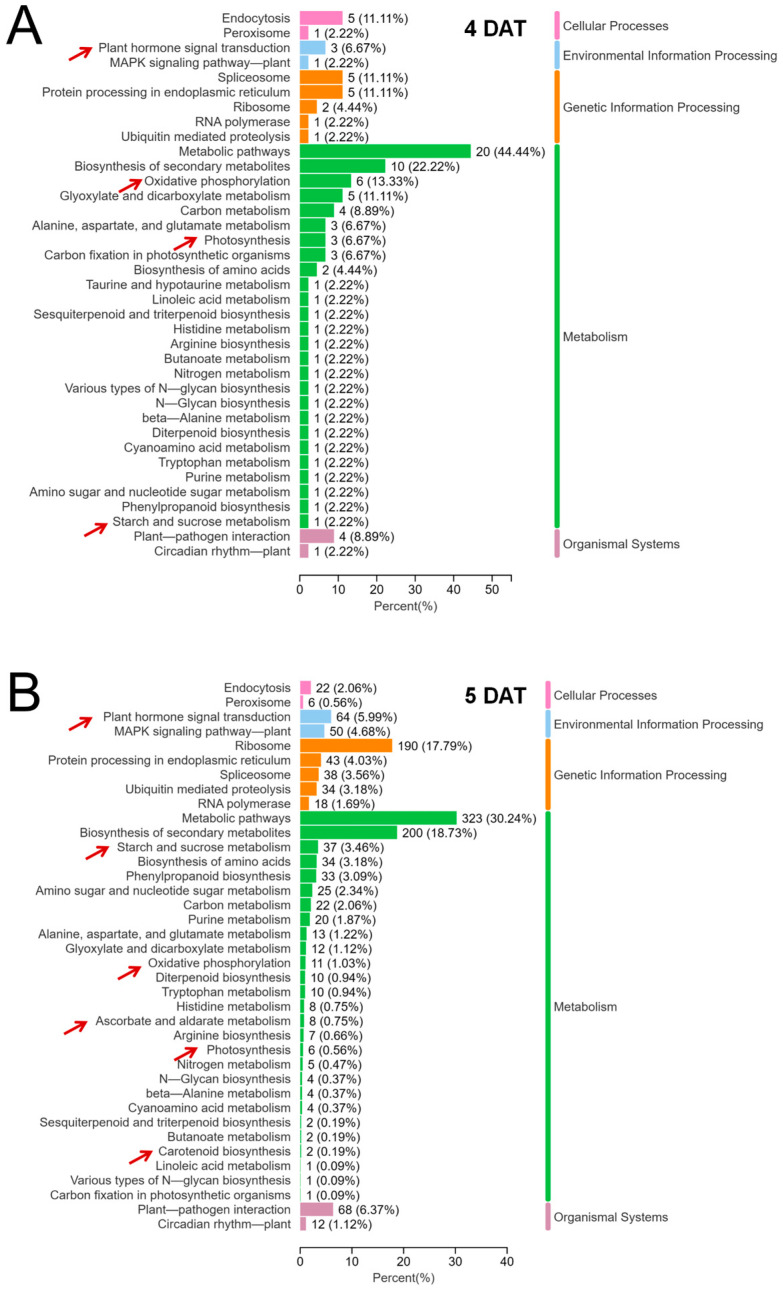
Metabolic pathway analysis of the differentially expressed genes from the transcriptome of the B03S leaf sheath. (**A**) Metabolic pathways of the differentially expressed genes in the 4-DAT leaf sheath. (**B**) Primary metabolic pathways of the differentially expressed genes in the 5-DAT leaf sheath. The arrow indicates metabolites related to glycometabolism and hormone-related pathways. The percentage indicates the proportion of differentially expressed genes in the pathway relative to the total number of differentially expressed genes.

According to the regulatory role of ABA and energy metabolism during the emergence of zebra traits, transcriptome analysis of the leaf sheath was further carried out in B03S to elucidate the underlying molecular basis and network. The results revealed 46 million clean reads, which overlapped with more than 92% of the rice genome. Significantly differentially expressed genes (DEGs) between the DT and ST leaf sheaths were selected according to the criteria FC ≥ 1.0 and *p* value ≤ 0.05. Before the emergence of zebra traits, a total of 127 DEGs, including 86 upregulated and 41 downregulated DEGs, were identified in the leaf sheath of B03S at 4 DAT under DT compared with ST. Moreover, 3256 DEGs, including 2324 upregulated and 932 downregulated, were identified in the sheath at the time of zebra trait emergence at 5 DAT. According to the results of our KEGG analysis, these DEGs were enriched in oxidative phosphorylation, photosynthesis, starch and sucrose metabolism, plant hormone signal transduction, ascorbate and aldarate metabolism, and carotenoid biosynthesis, which are mainly glucose metabolism pathways and hormone-related pathways (Figure 4).

**Figure 5:**
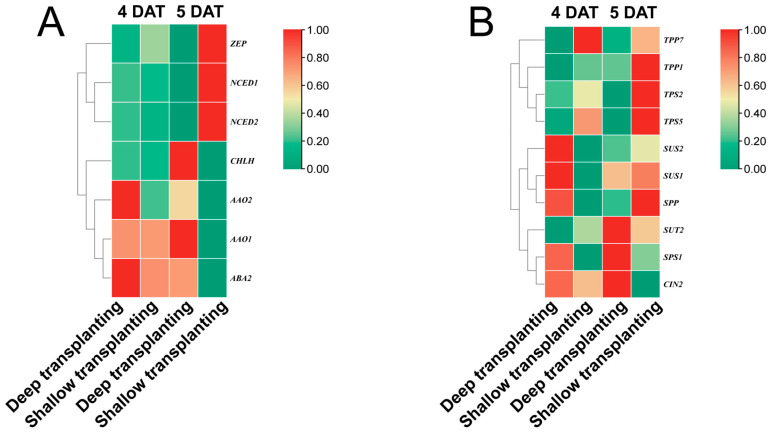
Heatmap analysis of gene expression involved in ABA synthesis and sucrose metabolism pathways in the leaf sheaths of B03S. (**A**) Heatmap analysis of gene expression in the ABA synthesis pathway. (**B**) Heatmap analysis of gene expression in the sucrose metabolism pathway. The data were normalized and transformed to the leaf sheath of B03S (*n* = 3).

Importantly, among these differentially expressed genes, we screened seven key genes involved in ABA metabolism (Figure 5A) and ten key genes involved in sucrose metabolism (Figure 5B). The expression of the gene *abscisic aldehyde oxidase 1* (*AAO1*), which encodes an enzyme that direct catalyzes ABA synthesis, was significantly increased in the fifth deep transplantation (DT-5) sample, which is consistent with the observed changes in the ABA content in the leaf sheath. These results suggest that ABA metabolism and sucrose metabolism may be involved in regulating secondary metabolism at the transcriptional level during B03S zebra trait formation. In addition, in the ABA metabolic pathway, the expression level of the *xanthoxin dehydrogenase 2* (*ABA2*) gene was 5.08 in the DT-5 sample, which was 1.69 times greater than that in the fifth shallow transplantation (ST-5) sample, and the expression of the *protoporphyrin IX magnesium chelatase* (*CHLH*) gene was 0.82 in the DT-5 sample, which was 3 times greater than that in the ST-5 sample.

### 2.5. Evaluation of the Effectiveness of Foliar Spraying

**Figure 6:**
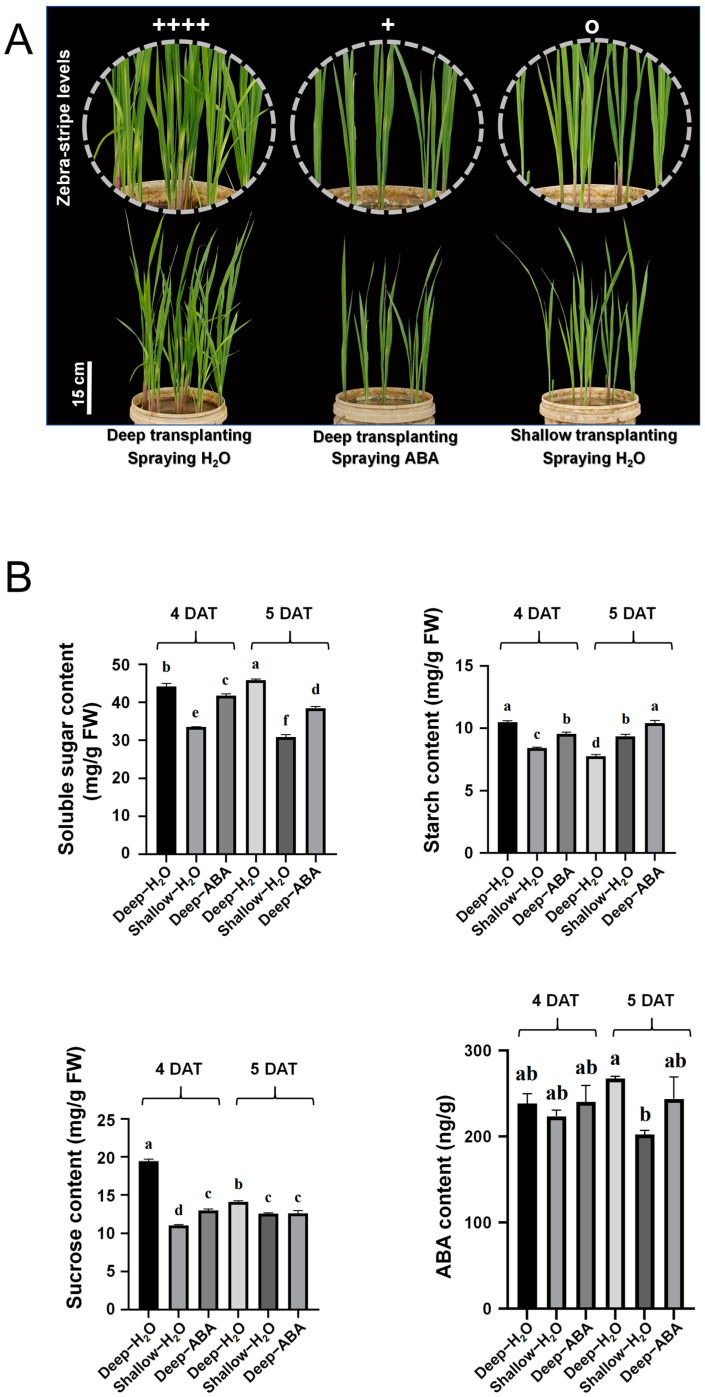
Effect of spraying ABA on the striped leaves of B03S plants under dark shading conditions. (**A**) Recovery of striped leaves under dark shading treatments of the 17-DAT leaf sheath by spraying ABA. (**B**) Starch, soluble sugar, sucrose, and ABA contents of the leaf sheath. Plus signs (+) indicate zebra stripe levels in B03S; o indicates full-green leaves. Bars represent means plus the standard error (*n* = 3). Means followed by the same letter are not significantly different (*p* < 0.05), as determined by Duncan’s multiple range test.

To elucidate the role of ABA and sucrose in the emergence of the zebra trait, we treated B03S plants with exogenous ABA, sucrose, and other nutrient substances during the dark shading treatment of the leaf sheath and studied their zebra stripe leaf characteristics. Both foliar spray treatments with ABA and sucrose delayed the generation of zebra leaf traits at 6 DAT and 7 DAT (Supplementary Figure S1A,B). During the period with obvious zebra leaf traits, foliar spray treatment with ABA restored the plants to a normal green color at 17 DAT, greatly decreasing the duration of zebra trait persistence (Figure 6A).

In general, foliar spraying could not completely eliminate the effects on zebra traits, and spraying ABA could not only alleviate the effects on zebra traits but also had an obvious effect on the recovery of zebra traits. With respect to the different shading treatments and different developmental stages of the zebra leaves, the effects of the spraying treatments with seven different solutions were slightly different. In the DT treatment of B03S seedlings in the third leaf stage, all the spraying treatments had some recovery effect on the appearance of zebra leaf traits, among which the groups sprayed with ABA, a mixture of ABA, brassinosteroid and KH_2_PO_4_, and sucrose exhibited stronger effects than the other groups at 6 DAT and 10 DAT (Supplementary Figure S1A). The foliar spray treatment had some effect on the recovery of zebra leaf traits. Compared with those in the CK group, the zebra leaf characteristics in the three spraying groups recovered significantly at 17 DAT (Supplementary Figure S1A). All the spraying treatments also had some effect on the appearance of zebra leaf traits under shading in B03S seedlings in the fifth leaf stage, among which the groups treated with ABA, a mixed solution of brassinosteroids and KH_2_PO_4_, and a mixed solution of trace elements exhibited better effects than the other groups at 7 DAT and 10 DAT (Supplementary Figure S1B). In the three spraying groups, the zebra leaf characteristics recovered significantly at 18 DAT (Supplementary Figure S1B).

### 2.6. High ABA Levels Inhibit Sucrose Accumulation in the Leaf Sheath under Dark Shading

According to the above results, the zebra stripe leaf trait of B03S recovered better after foliar spraying of ABA. To examine carbohydrate metabolism within the sheath during this period, changes in sugar and ABA contents in the sheaths of B03S plants were analyzed at the biochemical level.

After deep transplantation (DT), the soluble sugar content of the shaded part of the underground leaf sheath increased to 1.32 and 1.49 times greater than that of the photosensitive part after shallow transplantation at 4 DAT and 5 DAT, respectively. After ABA was sprayed on the DT materials, the soluble sugar content in the shaded part of the underground leaf sheath decreased, and the soluble sugar content on the fourth and fifth days after DT was 1.06 and 1.19 times greater than that after ABA was sprayed, respectively (Figure 6B). The changes in the sucrose content were consistent with the changes in the soluble sugar content. Under deep transplantation, the sheath sucrose contents at 4 DAT or 5 DAT were 1.76 to 1.12 times greater than those under shallow transplantation. After ABA was sprayed, the sucrose content of the sheath decreased slightly compared with that of the DT sheath. However, the sucrose contents of sheaths after ABA spraying were also 1.12 to 1.50 times greater than those of plants subjected to shallow transplantation (Figure 6B).

The starch content of B03S DT decreased by 27% at 5 DAT compared with that at 4 DAT. At 5 DAT, the starch content of the ST material increased by 7% compared with that at 4 DAT. After we sprayed ABA on the DT material, the starch content in the shaded part of the underground leaf sheath increased by 7% at 5 DAT and 4 DAT (Figure 6B). Moreover, we found that in the deep transplantation treatment, the content of sucrose and starch in leaf sheath at 4 DAT was 1.38 times and 1.35 times that at 5 DAT, respectively. After ABA spraying, the content of sucrose and starch in leaf sheath at 4 DAT was reduced to 1.03 times and 0.92 times of that at 5 DAT, respectively (Figure 6B).

In DT-5, the ABA content in the leaf sheath significantly increased to 58.21 ng/g, which was 3.99 times that in ST-5 and 10.85 times that in DT-4 (Figure 3). This change trend was consistent with the ELISA results (Figure 6B). Moreover, we observed that the ABA content in the leaf sheath decreased by 8.89% after ABA spraying at 5 DAT (Figure 6B). That is, after ABA was sprayed, sugar accumulation in the shaded part of B03S plants tended to increase toward the photosensitive part of the aboveground leaf sheath. In other words, ABA can alleviate zebra traits by controlling sugar redistribution.

### 2.7. Verification of the Expression of Genes Involved in the ABA Pathway and the Sucrose Metabolic Pathway in the B03S Leaf Sheath

Based on the effects of ABA and sucrose metabolic pathway genes on zebra trait formation, gene expression was analyzed by qPCR. Based on the transcriptome analysis, seven key genes involved in ABA metabolism and ten key genes involved in sucrose metabolism were selected (Supplementary Table S2).

**Figure 7:**
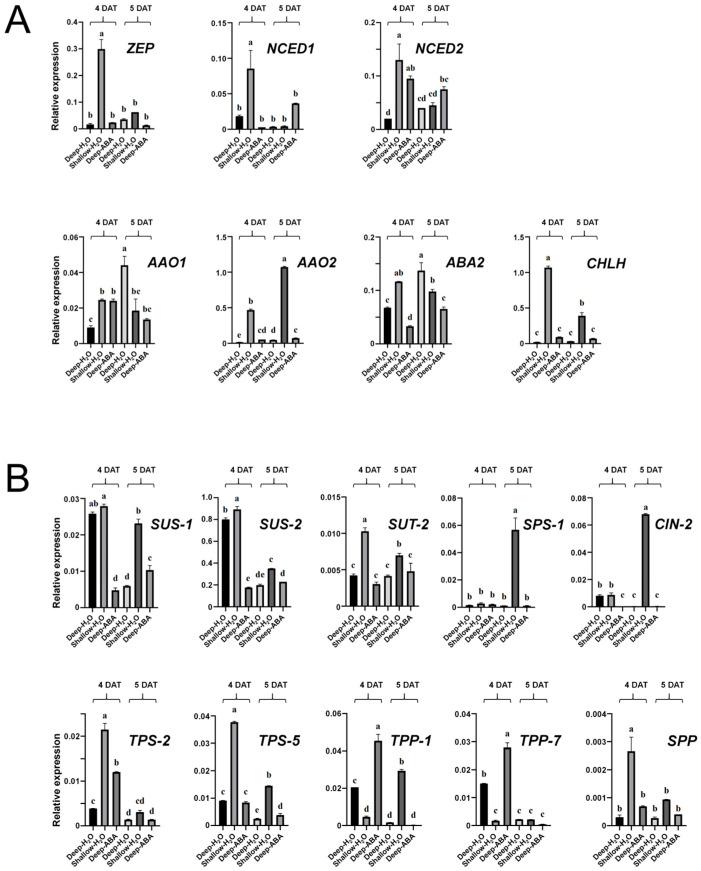
qPCR-based verification analysis of the expression of genes involved in ABA synthesis and sucrose metabolism-related pathways in the leaf sheath of B03S. (**A**) Analysis of the expression of seven genes involved in the ABA synthesis pathway. (**B**) Analysis of the expression of ten genes involved in the sucrose metabolism pathway. The internal reference gene used was *OsActin1*. Bars represent means plus the standard error (*n* = 3). Means followed by the same letter are not significantly different (*p* < 0.05) as determined by Duncan’s multiple range test.

Compared with ST-4, DT-4 showed downregulated expression of the *ZEP* gene and *CHLH* gene. Compared with ST-5, DT-5 showed downregulated expression of the *NCED1*, *NCED2*, and *CHLH* genes, while the expression of the *AAO1* and *ABA2* genes was upregulated (Figure 7A). The above gene expression patterns were consistent with the transcriptomic results (Figure 5), and the expression of the *AAO1* gene in DT-5 was significantly greater than that in the other treatments, which is consistent with our transcriptomic results. After ABA was sprayed on the leaves, both the *AAO1* gene and the *ABA2* gene were downregulated (Figure 7A), and we believe that these two genes are key genes involved in ABA synthesis in B03S leaf sheaths. Compared with ST-4, DT-4 showed downregulated expression of the *SUT2*, *TPS2*, and *TPS5* genes. Compared with ST-5, DT-5 showed downregulated expression of the *SUS1*, *SUS2*, *TPS2*, *TPS5*, and *TPP1* genes (Figure 7B), which is consistent with the transcriptomic results (Figure 5). The *SUS1* gene was upregulated 2.88-fold in ST-5 compared with DT-5, and after spraying ABA, the expression was downregulated 0.81-fold in ABA-4 compared with DT-4 and upregulated 0.73-fold in ABA-5 compared with DT-5. *SUS2* was upregulated 0.77-fold in ST-5 compared with DT-5, and after spraying ABA, the expression was downregulated 0.78-fold in ABA-4 compared with DT-4 and upregulated 0.15-fold in ABA-5 compared with DT-5. The *SUT2* gene was upregulated 1.43-fold in ST-4 versus DT-4 and upregulated 0.69-fold in ST-5 compared with DT-5. The *SPP* gene was upregulated 7.80-fold in ST-4 compared with DT-4 and upregulated 2.50-fold in ST-5 compared with DT-5 (Figure 7B). The expression trends in the above genes were consistent with the transcriptomic results. Moreover, we found that in the deep transplantation treatment, the expression levels of the *SUS1* and *SUS2* genes in the leaf sheath at 4 DAT were upregulated by 3.26 times and 3.04 times, respectively, compared with 5 DAT; after we sprayed ABA, the expression levels of the *SUS1* and *SUS2* genes in the leaf sheath at 4 DAT were downregulated by 0.54 times and 0.23 times, respectively, compared with 5 DAT (Figure 7B).

## 3. Discussion

In this study, the B03S material exhibited transverse discontinuous leaf greening, which we named the zebra trait. Previous studies have shown that this mutant trait is controlled by a pair of recessive genes and can be stably inherited. The characteristic leaf color phenotype of B03S is an albino trait, resulting in striped leaves displaying yellow–green intermittent stripes (Figure 1C). The mutant showed the zebra trait only when the transplantation depth exceeded 4 cm (Figure 2A). B03S is a photoperiod-affected PTGMS rice strain, so the temperature after transplantation affects the degree and duration of zebra leaf trait expression. Low temperature can shorten the occurrence time of the mutant phenotype, while high temperature is conducive to the development of mutant phenotype. However, excessive temperature can cause the trait to weaken or even not occur. In this study, the direct cause of striped leaf formation in B03S was found to be leaf sheath shading treatment. Deep transplantation and leaf sheath outer foil treatment were carried out through pot experiments (Figure 1A,B). After treatment, two to three new leaves exhibited zebra traits, and the zebra traits persisted for approximately 35 days. The results of the correlation analysis with photosynthesis showed that the photosynthetic capacity and photosynthetic products of the leaves of the seedlings with zebra leaf traits decreased, and the situation improved after the restoration of leaf color.

The contents of chlorophyll a and chlorophyll b in zebra leaves decreased significantly according to the results of photosynthetic pigment determination, indicating that zebra leaves exhibit total chlorophyll deficiency, and the results of electron microscopy showed that the chloroplast structure was obviously abnormal, especially the membrane structure on the matrix thylakoid, which was obviously incomplete. There was obvious sugar deficiency in the albino part of the zebra seedlings, and the expression of genes related to sugar transport decreased significantly. Since leaf sheath shading is the direct cause of zebra trait induction, we focused on sugar changes in the leaf sheath in this study. Sucrose phosphorylase (SPP) catalyzes the final step of the sucrose biosynthesis pathway, in which sucrose-6-phosphate is formed by sucrose phosphate synthase (SPS). SUTs (sucrose transporters) transport synthetic sucrose to vesicles or out of cells. After deep transplantation, the leaf sheath genes *SUT2*, *SPS1*, and *SPP* were significantly downregulated (Figure 7B). It is concluded that the leaf sheath changes from a source organ to a sink organ after leaf sheath shading and that sucrose needs to be transported from old leaves. During shallow transplantation, new leaves can be supplied with sucrose by leaf sheaths and old leaves, while during deep transplantation, the sucrose allocated to new leaves cannot supply normal growth, so there is obvious sugar deficiency in the albino part of zebra seedlings. This was in accord with the result of our determination of sucrose content in the leaf sheath. After the deep transplanting treatment, sucrose accumulation occurred in the basal leaf sheath on the DT-4 before the new leaves with the zebra character appearance in the B03S rice plants (Figure 6B).

During deep transplantation, we observed that all the spraying treatments had certain effects on zebra leaf traits, among which the three spraying groups (ABA, ABA+BR+P, and sucrose) exhibited better effects than did the other groups, while under leaf sheath shading, the spraying groups (ABA, Fe+Zn+Mg, and BR+P) exhibited better effects on zebra leaf traits. This may be related to the difference between the two treatment periods. For the deep transplantation treatment, we chose rice plants with robust growth in the three-leaf stage, while for leaf sheath shading, we chose the more robust five-leaf-stage rice; leaf sheath shading of rice in the three-leaf stage had a greater impact on the growth of rice. The zebra leaf traits differed between the deep transplantation group and the leaf sheath shading group. The B03S leaves in the deep transplantation treatment were more in line with the transverse interrupted loss-of-greening-related stripe traits of zebra leaves, and almost all the new B03S leaves in the leaf sheath shading treatment were yellow (Supplementary Figure S1A,B). This may be because the leaf sheath shading treatment is more harmful to B03S plants. Notably, ABA+BR+P had a greater effect on zebra traits in the deep transplantation treatment, while it had almost no effect in the leaf sheath shading treatment, which may be related to the different treatment periods and treatment methods. The plants in the deep transplantation group were in the three-leaf stage, the plants in the leaf sheath shading group were in the five-leaf stage, and the plants in the deep transplantation group exhibited less damage than did those in the leaf sheath shading group. In the leaf sheath shading group, the effects of ABA and BR+P spraying alone on zebra traits were obvious, but there was no effect after combined spraying, possibly because ABA and BR have certain antagonistic effects. We believe that ABA plays a major role in deep transplantation along with ABA+BR+P spraying, while in the leaf sheath shading group, the effect of the same concentration of ABA on B03S was not obvious.

In most plants, ABA induces premature leaf senescence, and studies have shown that the rice NAM/ATAF1/2/CUC2 (NAC) transcription factor ONAC054 plays an important role in ABA-induced leaf senescence. When *ONAC054*-overexpressing plants were floated on 3 mM 2-Morpholinoethanesulphonic acid (MES) buffer (pH 5.8) containing 100 μM ABA and incubated for the indicated periods, the *ONAC054*-overexpressing lines showed early leaf yellowing under dark- and ABA-induced senescence conditions [20]. ABA also plays an important role in regulating photosynthesis under stress. Under Polyethylene glycol (PEG) stress, exogenous ABA application significantly enhanced the recovery of the net photosynthetic rate, stomatal conductance, and transpiration rate in upland rice (resistant to drought stress), with increased expression of ABA-associated synthetic genes. In the chloroplast genes *OsPsbA*, *OsPsbD1*, and *OsPsbD2* encoding the major functional proteins D1 and D2 of PSII, it was found that the three genes of upland rice and lowland rice (susceptible to drought stress) had different responses to exogenous ABA treatment compared with PEG stress. In addition, the application of ABA resulted in a significantly increased expression level of *OsPsbD1* and *OsPsbD2* in upland rice, with a significantly reduced level of *OsPsbA* in lowland rice [21]. ABA did not repress the transcription of *psbD* or a few other genes and even increased *psbD* mRNA levels under certain conditions. Research shows that ABA affects the expression of chloroplast genes differentially, and when enhanced by light, the expression patterns of chloroplast genes are different at different sampling segments and times under exogenous ABA treatment [22]. In this study, we adopted a foliar spray of 100 μM ABA at 6 pm, avoiding the time of sunshine. Under the ABA treatment, the intermittent leaf discoloration characteristics caused by shading of the basal leaf sheath of B03S were significantly restored, and the time maintaining zebra leaf traits was shortened. We believe that this result is related to the different treatment methods and treatment times. Compared with the incubation in ABA solution, spraying leaves with ABA has less effect on plant growth, and the effect of ABA is mainly reflected in the resistance to adversity. Under a normal growth environment, long-term incubation in ABA solution is an adverse condition for plants. We think that exogenous spraying with the ABA solution enhanced the plant’s resistance to the stress of shading the base leaf sheath and reduced the synthesis of endogenous ABA in the plant. At the same time, ABA regulated sucrose metabolism by inhibiting the expression of the *SUS* gene and inhibited plant growth by regulating glycometabolism level. This allows for more sucrose synthesized by the old leaves to be transported to the new leaves. The striped leaf character had an obvious recovery effect due to re-balance of sugar supply and demand in rice. Since the effects of exogenous ABA on chloroplast gene expression vary with different varieties, treatment methods, treatment times, and sampling segments, we did not discuss the effects of exogenous ABA on chloroplast gene expression in this paper.

Xanthoxin synthesized in the plastid is subsequently transported into the cytoplasm to catalyze the formation of abscisic aldehyde by the short chain dehydrogenase encoded by *ABA2* and abscisic aldehyde by abscisic aldehyde oxidase (AAO)-mediated dehydrogenation to generate ABA. In rice, an ABA-deficient mutant, *aba2*, showed reduced ABA content [23]. These results indicated that the change in *ABA2* gene expression had an effect on ABA synthesis. Studies have shown that *AAO1* and *AAO4* in Arabidopsis seeds can partially promote ABA biosynthesis in *aao3* mutant seeds [24]. *Arabidopsis thaliana* AAO3 catalyzes the oxidation of abscisic aldehyde to ABA. *AAO3* knockout mutants (*aao3*) had lower ABA content than WT [25]. In response to drought stress, ABA accumulation levels in peanut leaves agree well with the upregulated expressions of ABA-producing genes *AhAAO2* [26]. In this study, the expression of seven ABA biosynthesis genes in the leaf sheath were analyzed not only by transcriptome sequencing but also by real-time quantitative PCR verification. It was found that both the *ABA2* and *AAO1* genes were upregulated in DT-5 (Figure 7A), and the ABA content significantly increased in DT-5 (Figure 3). These results indicated the expression levels of the two key *ABA2* and *AAO1* genes are closely related to the ABA content in the B03S sheath. *ABA2* and *AAO1* are the key genes downstream of ABA synthesis. Many previous studies have shown that ABA plays an important role in resistance to stress. Currently, most research focuses on ABA as a signaling molecule in plants in response to environmental stress, including abiotic stresses such as drought, salt, osmotic and cold stresses [27,28,29,30]. In addition, ABA regulates plant responses to viral infections through the following two different defense mechanisms: the antiviral RNA silencing pathway and callose accumulation [31]. ABA can regulate the accumulation of soluble sugars by affecting the transcription levels of genes associated with sugar synthesis and transport in plants [32]. Soluble sugars can participate in the response to abiotic stress by influencing osmotic potential [33]. Soluble sugars respond to water stress and salt stress [34,35]. In *Arabidopsis thaliana*, the soluble sugar content increases under drought stress [36]. After deep transplantation, leaf sheath ABA induced the accumulation of soluble sugars (Figure 6B), which further enhanced the stress resistance of the plants. In the leaf sheaths of plants subjected to deep transplantation, the components of the energy metabolism pathway were mainly PPP metabolites (Figure 2B–D), and both the ATP content and energy charge were greater in these plants than in those subjected to shallow transplantation (Figure 2E). The high energy charge inhibited the production of ATP and promoted the utilization of ATP at the same time, that is, it promoted anabolic metabolism [37]; therefore, we can assume that the energy in deeply transplanted leaf sheaths is mainly used to resist the stress of basal leaf sheath shading. Research has found that the application of exogenous ABA has contrasting effects on arbuscular mycorrhizal (AM) and non-AM plants. The addition of exogenous ABA considerably enhanced the ABA content in the shoots of non-AM plants. In contrast, the addition of exogenous ABA decreased the content of ABA in the shoots of AM plants [38]. After exogenous spraying of ABA, we found that the ABA content decreased in leaf sheaths, and the expression of ABA synthesis genes *ABA2* and *AAO1* was downregulated, indicating that exogenous ABA can lead to a decrease in endogenous ABA synthesis in leaf sheaths. We believe that exogenous ABA enhances the resistance of B03S to shading at the base of leaf sheaths, and the decrease in endogenous ABA synthesis in plant leaf sheaths reduces the demand for sucrose in leaf sheaths, resulting in more sucrose flowing to new leaves. The *CHLH* gene plays an important role in the chlorophyll synthesis pathway and can encode the H-subunit of magnesium chelatase. Magnesium chelatase is an enzyme of the magnesium branch of plant chlorophyll synthesis and is composed of the following three subunits: I (ChlI), D (ChlD), and H (ChlH) [39]. To date, research on the *CHLH* gene has focused mainly on the signal transduction function of ABA and chlorophyll synthesis [40,41,42]. CHLH can bind to ABA and mediate ABA signaling through WRKY domain transcriptional suppressors, confirming that CHLH is a receptor for ABA [40]. In this study, deep transplantation resulted in significantly downregulated expression of the *CHLH* gene (Figure 7A), and B03S belonged to the total chlorophyll deficiency type. After deep transplantation, the synthesis of chlorophyll a and chlorophyll b in new leaves was affected, which was consistent with previous results from our research group. When plants subjected to deep transplantation were subjected to ABA spraying, the expression of the *CHLH* gene increased (Figure 7A), which alleviated the appearance of zebra leaf traits in B03S.

After exogenous ABA spraying, the sucrose content in the leaf sheath decreased (Figure 6), indicating that the amount of sucrose transported from the old leaves to the leaf sheath decreased, resulting in more sucrose being transported to the new leaves. Moreover, exogenous ABA spraying improved the stress resistance of the plants. The plants did not need to expend too much energy to improve their resilience. Sucrose synthase (SUS) can reversibly catalyze the conversion of sucrose and nucleoside diphosphate (NDP) to fructose and nucleoside diphosphate glucose (NDPG) [43]. SUS may play another, less studied role in the development of the shoot apical meristem (SAM). The SAM receives Suc from the phloem, and there is evidence that *SUS* is expressed in the SAM. Functional studies also suggest that *SUS* genes play significant roles in the SAM and in early leaf development. Transgenic tomato lines with suppressed *SlSUS1*, *SlSUS3*, and *SlSUS4* exhibited abnormal cotyledons and leaf morphology [44]. More importantly, sucrose synthase can catalyze the decomposition and synthesis of sucrose with low energy consumption and can quickly transport sucrose from the “source” organ to the “sink” organ; the entire physiological and biochemical process does not require the participation of other biochemical enzymes [45]. It was found that ABA can control sucrose metabolism by mediating *SUS* gene expression [46]. After exogenous ABA application, the expression levels of *SUS1* and *SUS2* significantly decreased on the 4th day, and the expression levels of *SUS1* and *SUS2* significantly decreased in DT-5 compared with DT-4. However, after exogenous ABA application, the expression levels of *SUS1* and *SUS2* were increased in ABA-5 compared with ABA-4 (Figure 7B), indicating that the leaf sheath showed a growth delay after ABA application. In summary, we believe that after exogenous ABA application, plants have more energy for the formation of new leaves, so shallower and fewer zebra stripes occur in new leaves.

ABA, first discovered in the 1860s, is a weak C_15_ acid and a sesquiterpenoid compound composed of isoprene [47]. It is present in all vascular plants and plays a crucial role in the resistance of plants to various stresses. ABA exists in the following two forms: 2-cis and 4-trans. Cis-ABA has physiological effects, while trans-ABA has very weak physiological activity. ABA is widely found in plants and is produced by the cleavage of C_40_ carotenoids, which undergo continuous isomerization and desaturation to produce all-trans lycopene. Then, lycopene beta cyclization occurs to form beta-lycopene, and further hydroxylation occurs to form zeaxanthin. The synthesis of violaxanthin is catalyzed by zeaxanthin epoxidase (ZEP). Violaxanthin is converted to neoxanthin by the action of neoxanthine synthetase (NSY). Violaxanthin and neoxanthine are then catalytically converted to xanthoxin by NCED, which is recognized as the rate-limiting step in the de novo synthesis of ABA. The cleavage reaction of xanthoxin is mediated by *ABA2*-encoded short-chain dehydrogenase (SDR), AAO, and molybdenum cofactor (MoCo) to eventually generate ABA [48,49]. Because of the many forms of the ABA structure, it plays a crucial role in plant resistance. Stomata are crucial for gas exchange and transpiration in plants, and drought [50], darkness [51], high carbon dioxide levels [52], pathogen infection [53], and abiotic and biological threats may induce stomatal closure. Stomatal closure is the main process by which plant transpiration is reduced to prevent water loss, and ABA can play an important role in stomatal closure through Ca^2+^-dependent or Ca^2+^-independent pathways [54], thus improving the drought resistance of plants. ABA and ethylene may regulate and influence fruit color development through synergistic effects of genes related to the carotenoid, phenolic, and flavonoid synthesis pathways [55]. A deficiency in ABA in the tomato mutant *hp3* leads to an increase in the lycopene content in the fruit [56]. Current studies have shown that ABA can inhibit plant growth [57,58,59].

**Figure 8:**
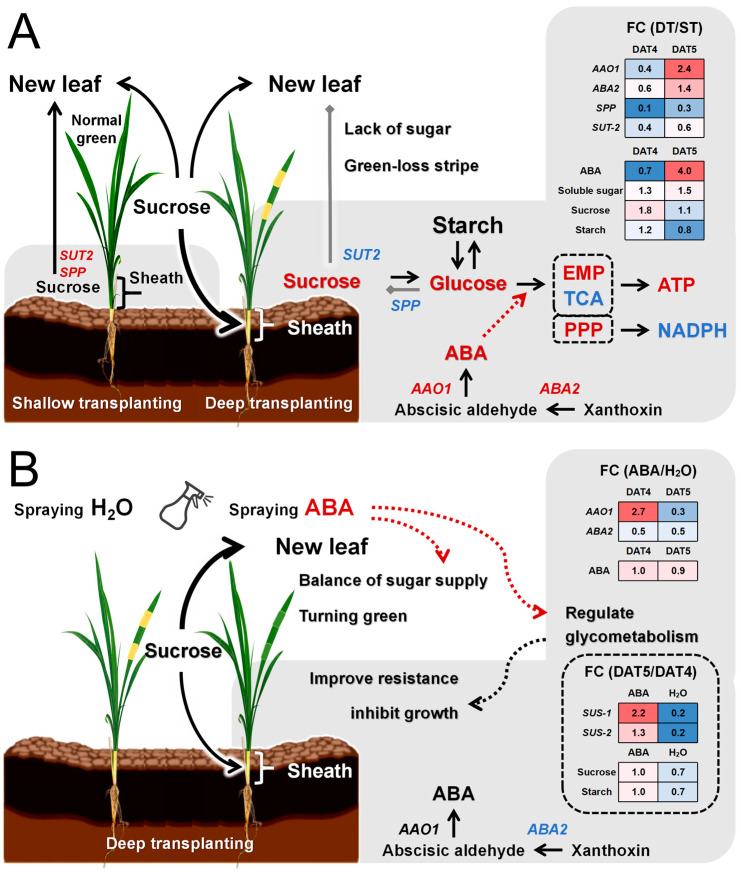
Diagram of endogenous ABA synthesis gene expression and glycometabolism of B03S under different processing conditions. (**A**) Contrast between shallow and deep transplantation, where the leaf sheath changes from a source organ to a sink organ, enhancing stress resistance by competing with new leaves for sugar. (**B**) Comparison between deep transplantation and deep transplantation with exogenous ABA application. After exogenous ABA application, the overall stress resistance of B03S plants improved, and the greening of new leaves was promoted by sugar redistribution.

We also observed that the leaf sheath sucrose content and starch content were significantly higher in DT-4 than in ST-4 (Figure 6B). This accumulation of sucrose and starch content in the leaf sheath was thought to be in preparation to sprout new leaves at 5 DAT, and we found that the sucrose and starch contents decreased significantly in DT-5 compared with DT-4. On the one hand, the new leaves need to supply energy from the old leaves; on the other hand, the plants have to resist the stress caused by shading the base leaf sheath, which is a collaborative process. After spraying ABA, the content of sucrose and starch did not change significantly in ABA-5 compared to ABA-4 (Figure 6B). We believe that exogenous ABA mainly plays two roles here, one is to enhance the resistance of plants to the stress caused by shading the base leaf sheath, and the other is to delay the growth of plants, so that plants can be more fully prepared for the growth of new leaves (Figure 8B).

The main physiological functions of the rice leaf sheath include supporting leaves, providing nutrients, and storing substances. Some studies have shown that the leaf sheath of rice sword leaves, like leaves, possesses Rubisco, which has carboxylation activity [60], participates in the Calvin cycle and CO_2_ fixation, has a certain photosynthetic, rate and can produce photosynthetic products. In this study, after the shading treatment of the B03S basal leaf sheath, the leaf sheath changed from the original source organ, providing sucrose for new leaves, to the sink organ, competing with new leaves, resulting in a sugar deficiency in the new leaves showing the zebra leaf trait (Figure 8A).

## 4. Materials and Methods

### 4.1. Plant Material and the Leaf Color Phenotype

The natural leaf color mutant B03S derived from the rice cultivar Efeng 1S (*Oryza sativa* L. ssp. *indica*) was crossed with the conventional cultivar 9311 (ssp. *indica*) for six generations [16]. The resulting mutant exhibited obvious loss of green stripes after deep transplantation. Specifically, the leaf sheaths and leaves of B03S plants exhibited albino stripes interrupting green stripes, which ultimately appeared as yellow–green intermittent stripes that resembled zebra stripes. This striped-leaf trait persisted on each new leaf, gradually returning to a full, normal green color, lasting for a total of approximately 35 days. In addition to occurring at the seedling stage, these zebra stripes could occur after deep transplantation throughout almost the entire vegetative growth phase and were mostly concentrated within new leaves and/or sheaths.

### 4.2. Phenotypic Analysis by Transplantation and Spraying Treatments

Pot experiments were performed at Huazhong Agricultural University in Wuhan (30°47′ N, 114°35′ E) under natural conditions from May to October in 2022 and 2023. B03S plants were sown and grown in pots (25 cm in diameter and 30 cm in height; 15 cm in diameter and 14 cm in height), with five plants in each pot.

Phenotypic analysis of B03S plants at the 3rd-leaf and 5th-leaf stages was carried out under shading and spraying treatments. First, two types of shading treatments involving the base leaf sheath were used to induce the formation of striped leaves as follows: (1) shallow and deep transplantation groups of 3rd-leaf-stage seedlings, in which plants were transplanted in pots with 2 cm and 5 cm of the leaf sheaths below the soil, and (2) an aluminum shading group of 5th-leaf-stage plants, in which plants in pots were covered with aluminum foil to a height of more than 5 cm, with non-transplanted plants used as a control. Moreover, spraying treatments with seven different solutions, including (1) H_2_O, (2) 2.64 mg/mL ABA (Yuanye, Shanghai, China), (3) 10 mg/mL sucrose solution (Yuanye, Shanghai, China), (4) 1 mg/mL trace element solution containing FeSO_4_, ZnSO_4_, and MgSO_4_, (5) 0.01 mg/mL brassinosteroid solution (Yuanye, Shanghai, China) and 0.3 g/mL KH_2_PO_4_ solution, (6) 2 g/mL urea + 1 g/mL sucrose + 0.3 g/mL KH_2_PO_4_, and (7) 2.64 mg/mL ABA solution + 0.01 mg/mL brassinosteroid solution + 0.3 g/mL KH_2_PO_4_, containing 0.5 µL/mL silwet L-77 surfactant (Yuanye, Shanghai, China), were applied to the plants in the deep transplantation group and the aluminum shading group to examine their effects on the striped leaf trait. The spraying time was 6 pm, and the aboveground part of each plant was sprayed. Spraying was stopped when the leaves were moist and dripping. The plants were sprayed once a day until the zebra leaf characteristics disappeared.

ABA was applied only to 3rd-leaf-stage seedlings under deep transplantation, and both spraying ABA and shallow/deep transplantation were considered for subsequent analysis. Leaf sheath material was sampled routinely according to the dynamic changes in the striped leaves. Before and after the emergence of the newest leaf, leaf sheath samples were collected 4 days and 5 days after transplantation (DAT), quickly immersed in liquid nitrogen, and placed in a centrifuge tube in a −80 ℃ cryogenic freezer until subsequent experiments were performed. As for the sampling time, a previous research group found that DT-4 was the day before the new leaves with zebra character were sprouted, and DT-5 was the time when the new leaves with zebra character were sprouted [16]. In terms of sampling methods, we clipped shallow transplantation aboveground leaf sheaths (light sensitive) and deep transplanting underground leaf sheaths (light shaded).

### 4.3. UPLC-MS/MS Analysis of Glycometabolism and ABA in the Leaf Sheath

Metabolomic analysis of glycometabolism in the leaf sheath of B03S plants at 4 DAT and 5 DAT was performed using ultra-performance liquid chromatography/tandem mass spectrometry (UPLC-MS/MS) [61], following the procedure described in our previous studies [16]. There were three biological replicates per treatment. The metabolomes were determined by MetWare (Wuhan, China) according to previously described methods [62].

All of the standards were purchased from Sigma–Aldrich (St. Louis, MO, USA), and a total of 80 target metabolites were detected (Supplementary Table S1). A total of 0.05 g of the sample powder was mixed with 500 µL of 70% methanol (Merck, Darmstadt, Germany) in water as the internal standard extractant and 300 µL of dichloromethane. The sample was vortexed for 3 min at 2500 r/min and centrifuged at 12,000 r/min for 10 min. Then, 200 μL of the supernatant was used for LC–MS analysis.

The sample extracts were analyzed by LC–ESI–MS/MS using an Acquity H-ClassD system (Waters, Manchester, U.K.) and a Qtrap 6500+ system (AB Sciex, Framingham, MA, USA). The analytical conditions for UPLC were as follows: the column used had dimensions of 2.1 mm, 100 mm, and 1.7 μm; the solvent system used was 10 mM ammonium acetate with 0.3% ammonium hydroxide and 90% (*v*/*v*) acetonitrile; the flow rate was 0.4 mL/min; the temperature was 40 °C; and the injection volume was 2 μL. Linear ion trap and triple quadrupole scans were acquired on a triple quadrupole-linear ion trap mass spectrometer (AB Sciex, Framingham, MA, USA). The analytical conditions were a source temperature of 550 °C and ion spray voltages of 5500 V and −4500 V. All the metabolites were detected by MetWare (Wuhan, China; http://www.metware.cn/ (accessed on 8 January 2024)) and quantified by Multiquant 3.0.3 software (AB Sciex, Framingham, MA, USA).

ABA in the leaf sheath was also measured via UPLC-MS/MS. There were three biological replicates per treatment. A total of 0.05 g of the sample powder was dissolved in 1 mL of methanol/water/formic acid (15:4:1, *v*/*v*/*v*), with internal standards (Olchemim Ltd., Olomouc, Czech Republic) added for quantification. A total of 25 target metabolites were detected (Supplementary Table S1). Then, the supernatant was used for qualitative and quantitative analysis.

### 4.4. Transcriptome Sequencing of the Leaf Sheath

Total RNA was extracted from the leaf sheaths of shallowly transplanted and deeply transplanted plants using RNAiso Plus Total RNA Extraction Reagent (Takara, Beijing, China) following the manufacturer’s instructions, and RNA integrity was checked via an Agilent Bioanalyzer 2100 (Agilent Technologies, Santa Clara, CA, USA). RNA purity was checked using a NanoPhotometer spectrophotometer (Implen, CA, USA).

The cDNA libraries were sequenced on the Illumina sequencing platform by MetWare (Wuhan, China). The mRNA library was prepared from 1 µg of total RNA via a TruSeq sRNA Kit (Illumina, San Diego, CA, USA) and a cBot Clonal Amplification System (Illumina, San Diego, CA, USA). Sequencing was performed on an Illumina HiSeq 2500 (Illumina, San Diego, CA, USA) with three biological replicates.

All subsequent analyses were based on clean reads. The reference genome and its annotation files were downloaded from the designated website, HISAT v2.1.0 was used to construct the index, and the clean reads were compared to the reference genome. The number of fragments in a transcript is related to the amount of sequencing data (or Mapped Data), the length of the transcript, and the expression level of the transcript. In order for the fragment number to truly reflect the expression level of the transcript, the number of Mapped Reads in the sample and the length of the transcript need to be normalized. We used featureCounts v1.6.2 to calculate the gene alignment and then calculated the fragments per kilobase million (FPKM) value of each gene based on the gene length. FPKM analysis is currently the most commonly used method to estimate gene expression levels. DESeq2 (v1.22.1) was used to analyze differential expression between the two groups, and the *p* value was corrected using the Benjamini–Hochberg method [63]. The corrected *p* value and |log2(fold change)| were used as the thresholds for significant differences in expression. We used the clusterProfiler R package to test the statistical enrichment of differentially expressed genes in Kyoto Encyclopedia of Genes and Genomes (KEGG) pathways (http://www.genome.jp/kegg/ (accessed on 8 May 2023)).

### 4.5. Measurements of Sucrose, Starch, and ABA Contents in the Leaf Sheath

To confirm the changes in sugar and hormone accumulation, the concentrations of soluble sugars, sucrose, and starch were measured at the biochemical level under the three treatments of shallow and deep transplantation and ABA spraying in the sheath. The determination of soluble sugar levels was performed using a Plant Soluble Sugar Content Testing Kit (Comin, Suzhou, China) according to the principles of the anthrone colorimetric method [64]. Subsequently, the isolated starch was hydrolyzed via acid hydrolysis, and the starch content was determined using a Starch Content Testing Kit (Comin, Suzhou, China) according to the anthrone colorimetric method.

The sucrose content was measured using a Sucrose Content Testing Kit (Comin, Suzhou, China). In this process, the sample was coheated with alkali to destroy the reducing sugars. Then, under acidic conditions, sucrose was hydrolyzed to produce glucose and fructose, which further reacted with resorcinol to produce colored substances with characteristic absorption peaks at 480 nm in a Bio Tek Epoch instrument (Agilent Technologies, Santa Clara, CA, USA). The above experimental steps were performed as described by the manufacturer. There were three replicates per treatment.

An Abscisic acid Enzyme-linked Immunoassay Kit (Fankewei, Shanghai, China) was used to measure the ABA content. The underlying principle for determining the ABA level in plant samples involved the double-antibody sandwich method. Purified plant ABA antibodies were coated on microporous plates to prepare solid-phase antibodies. ABA was added to the micropores coated with the mABs and then combined with HRP-labeled ABA antibodies to form an antibody–antigen–enzyme-labeled antibody complex. After thorough washing, the substrate TMB was added for color development. The TMB reaction was catalyzed by HRP to obtain a blue color, which was converted to the final yellow color under the action of acid. The depth of color was positively correlated with ABA in the sample. The absorbance (OD) was measured at a wavelength of 450 nm by an enzymoleter, and the ABA concentration was calculated from the standard curve.

### 4.6. Real-Time Quantitative PCR-Based Validation of Gene Expression in the Leaf Sheath

To validate the gene expression trends, the relative expression levels of genes related to ABA synthesis and sucrose metabolism, such as *OsZEP*, *OsNCED1*, *OsNCED2*, *OsAAO1*, *OsABA2*, *OsAAO2*, *OsCHLH*, *OsSUS1*, *OsSUS2*, *OsSUT2*, *OsCIN2*, *OsSPS1*, *OsSPP*, *OsTPS2*, *OsTPS5*, *OsTPP1*, and *OsTPP7*, were measured under the three treatments in the sheath by real-time quantitative PCR (RT–qPCR). The primers for the seventeen genes were designed according to the NCBI database and synthesized by Bioengineering Co., Ltd. (Shanghai, China; Supplementary Table S2).

Total RNA was extracted using an RNAprep Pure Plant Kit (Tiangen, Beijing, China) as described by the manufacturer. For qPCR-based verification of gene expression, 0.25 µL of RNA from each sample was used, with three biological replicates included. First-strand cDNA was synthesized using an RT mix with DNase (US Everbright Inc., Suzhou, China). qPCR analysis was then performed using SYBR Green qPCR Master Mix (Bio-Rad, Hercules, CA, USA) on a QuantStudio 6 Flex Instrument (Applied Biosystems, Foster, CA, USA). The relative expression was analyzed according to the 2^−∆CT^ method [65]. The expression levels were normalized against the expression level of *Actin1* (*Os03g0718100*), which served as a reference gene.

### 4.7. Statistical Analyses

The values were subjected to analysis of variance (ANOVA), and significant differences were analyzed using Prism (GraphPad Software, San Diego, CA, USA), and the ANOVA accompanied by Duncan were analyzed using SPSS Statistics 25 (IBM, Armonk, NY, USA). Images were prepared with PowerPoint software (Microsoft Corp., Redmond, WA, USA) and Photoshop software (Adobe, San Jose, CA, USA). The transcriptome heatmap was generated with TBtools [66]. Field photos were taken by a digital camera (EOS RP, Canon, Japan).

## 5. Conclusions

In conclusion, we confirmed that leaf sheath shading induced zebra leaf traits in B03S. After leaf sheath shading, the leaf sheath changed from the original source organ, providing sucrose for new leaves, to the sink organ, competing with new leaves for sucrose, resulting in serious sugar deficiency in new leaves. We observed the accumulation of sucrose and starch contents in the leaf sheath in DT-4 to prepare for the sprouting of new leaves, and then their sucrose and starch contents decreased significantly in DT-5 by deep transplantation. Physiological metabolism in the leaf sheath occurs mainly to resist the stress caused by basal leaf sheath shading. It manifests as the conversion of the energy metabolic pathway to the PPP, an increase in the energy charge and ATP content, and upregulated expression of *ABA2* and *AAO1*, the key genes involved in ABA synthesis, resulting in ABA accumulation and increased glucose levels. After exogenous ABA application, the resistance level of B03S plants to the stress caused by basal leaf sheath shading was increased, by inhibiting the expression of the *SUS* gene to regulate sucrose metabolism, with no significant changes in the leaf sheath sucrose and starch contents. This indicates that ABA may slow down plant growth by regulating glycometabolism levels, thus alleviating the sugar deficiency in the new leaves.

Taken together, the findings show that *ABA2* and *AAO1* regulate ABA synthesis, and ABA controls sucrose distribution through *SUS*. These results provide new ideas for further analysis of the mechanism underlying uneven leaf color distribution in nature.

## Data Availability

The data are contained within this article.

## Supplementary Materials

The following supporting information can be downloaded at https://www.mdpi.com/article/10.3390/plants13152090/s1: Figure S1: Effect of spraying sheaths of the striped leaves of B03S under the dark shading treatment. (A) Leaf phenotype of plants subjected to deep and shallow transplantation. (B) Leaf phenotypes of plants after aluminum foil covering, non-transplantation, and deep transplantation; Table S1: Linear equation of the standard curve and correlation coefficient of the internal standard; Table S2: Primer pair information of candidate genes.
