# Supplementary material for: Abscisic Acid Regulates the Occurrence and Recovery of the Striped Leaf Phenotype in Response to Lacking Light at the Base of Sheath in Rice by Modulating Carbohydrate Metabolism
Source: Plants (Basel). 2024 Jul 28;13(15):2090. doi: 10.3390/plants13152090 (PMC11314377; doi:10.3390/plants13152090)
Supplement: Supplementary file 1 [file plants-13-02090-s001.zip › Supplementary Figure S1.pdf]

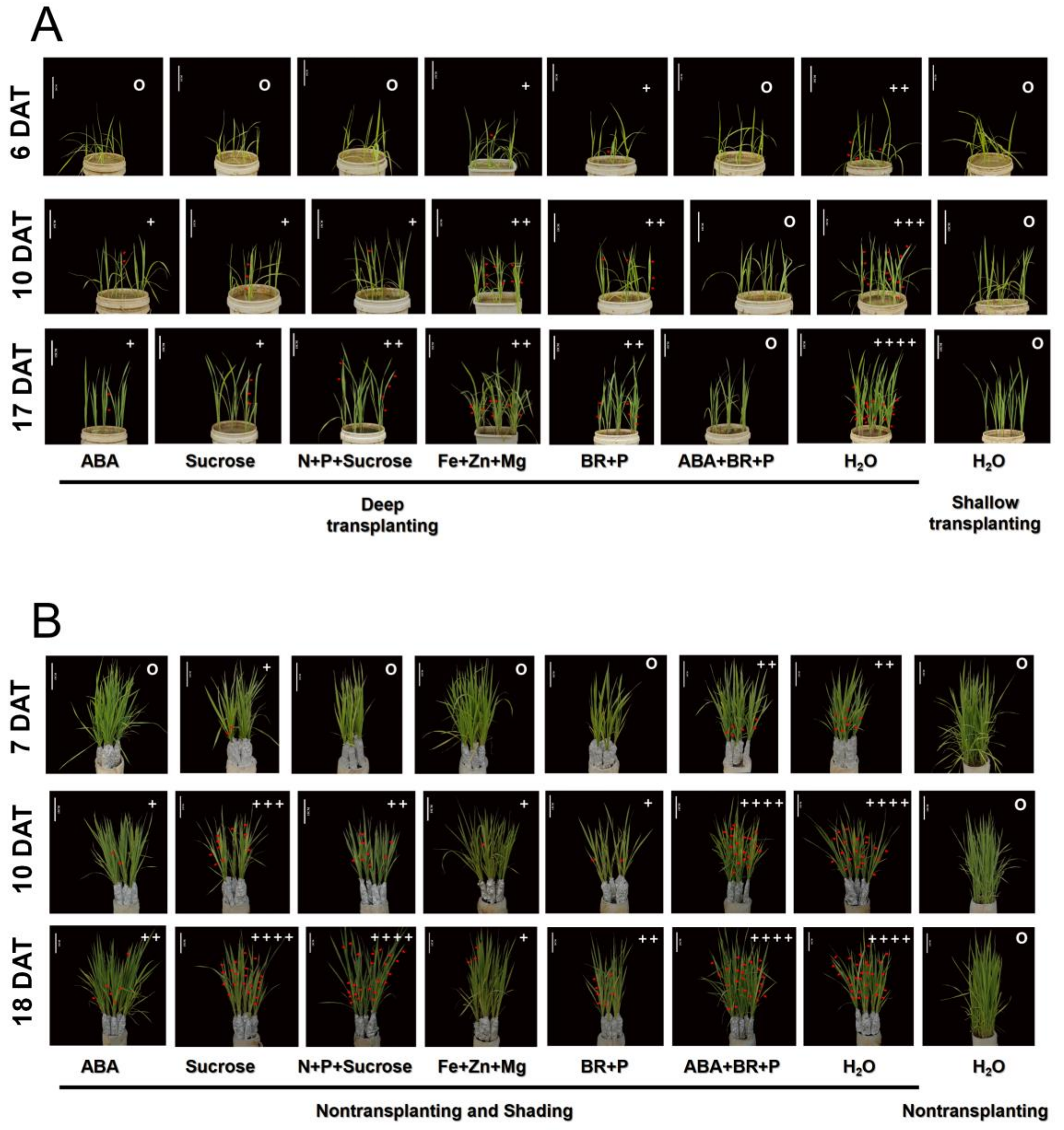

Supplementary Figure S1. Effect of spraying on sheaths of the striped leaves of B03S under dark shading treatment. (A) Leaf phenotype of plants subjected to deep and shallow transplantation. (B) Leaf phenotypes of plants after aluminum foil covering, nontransplantation, and deep transplantation.
